# The comparative results of myostatin introgression from donor Texel to recipient Ramlıç sheep with the aspects of growth, pre-, and post-slaughter carcass traits in the second backcross generation

**DOI:** 10.5194/aab-65-231-2022

**Published:** 2022-06-22

**Authors:** Mustafa Tekerli, Metin Erdoğan, Serdar Koçak, Koray Çelikeloğlu, Ebubekir Yazıcı, Özlem Hacan, Zehra Bozkurt, Mustafa Demirtaş, Samet Çinkaya

**Affiliations:** 1 Department of Animal Science, Faculty of Veterinary Medicine, Afyon Kocatepe University, Afyonkarahisar, Türkiye; 2 Department of Veterinary Biology and Genetics, Faculty of Veterinary Medicine, Afyon Kocatepe University, Afyonkarahisar, Türkiye; 3 Department of Obstetrics and Gynecology, Faculty of Veterinary Medicine, Afyon Kocatepe University, Afyonkarahisar, Türkiye

## Abstract

The aim of the study was to evaluate the growth, body and ultrasonic measurements and some carcass traits of purebred Ramlıç, Texel and crossbred (87.5 % Ramlıç and 12.5 % Texel) lambs in a marker-assisted introgression (MAI) project. The effects of some environmental factors such as genotype, sex, birth type, dam age, weaning age, and birth weight on growth, ultrasound measurements, and carcass traits were significant ( P<0.05 ). There were no significant differences between pure Ramlıç and its myostatin carrier ( +/- ) and non-carrier ( -/- ) second backcrosses (BC ${}_{2})$ for birth weight, daily live weight gain, and weaning weight. The BC ${}_{2}$ genotype ( +/- ) was statistically caught up with pure Ramlıç for wither height, body length, and rump width. Differences in the longissimus muscle depth indicated that the BC ${}_{2}$ ( +/- ) genotypes tended to be better for both pure Ramlıç and Texel lambs. Texel lambs were superior to Ramlıç in the aspect of some carcass characteristics such as leg length, cold right-half carcass weight, foreleg weight in the left-half carcass, and muscle weight in the left leg. BC ${}_{2}$ ( +/- ) lambs were in the middle of both breeds for the traits. BC ${}_{2}$ lambs carrying myostatin did not vary from Ramlıç morphologically. The new type of Ramlıç was also closer to Texel in the aspect of carcass characteristics. In this connection, improvement of indigenous breeds could be achieved by MAI without changing the essential characteristics. For the summary, please visit http://www.mustafatekerli.com (last access: 14 June 2022).

## 1 Introduction

Türkiye, with a population of 45 million heads of sheep, is a leading country in Europe. Increasing population and industrialization caused an intense demand for meat production in the country. However, boosting the number of sheep would not be sufficient for highly priced carcass parts and a profitable income. Urbanization and diminishing pastures require the use of technology in the sheep industry. In recent years, great activities on crossbreeding including molecular techniques have been emphasized for more production in livestock breeds. Marker-assisted introgression (MAI) may take place in the sheep industry to develop the characteristics of meat. Texel is one of the most known muscled sheep breeds. The single nucleotide polymorphism (g + 6723G > A) found in the 3 ${}^{\prime }$ UTR region of GDF8 gene in Texel sheep which is known as myostatin was reported to cause superior muscularity (Clop et al., 2006; Çelikeloğlu et al., 2021; Grasset et al., 2009; Johnson et al., 2005; Yalçın et al., 1978; Yayvan and Özkul, 2018). This mutation accelerates growth and increases muscularity in Texel and does not have a negative effect on the eating quality and intramuscular fat of the loin (Kijas et al., 2007). An introgression project was initiated to give a new dimension to Ramlıç's meat quality and quantity through the integration of the Texel myostatin mutation (g + 6723G > A). The detailed introductory information and $F_{1}$ and first backcross (BC ${}_{1}$ )-related results were articulated in our previous paper (Çelikeloğlu et al., 2022).

The study aims to evaluate the influence of myostatin mutation (g + 6723G > A) on morphometric and post-slaughter characteristics of BC ${}_{2}$ genotype in comparison with Ramlıç and Texel pure lambs.

## 2 Materials and methods

The animals were used in compliance with the rules of experimental animals ethical committee in Afyon Kocatepe University (decision no. 49533702-26). A total of 103 pure Ramlıç ewes ( -/- ) were mated with 24 BC ${}_{1}$ ( +/- ) ram lambs by flock mating to obtain more lambs carrying myostatin mutation, and 118 BC ${}_{2}$ (12.5 % Texel, 87.5 % Ramlıç) lambs were produced. Ramlıç and Texel contemporary lambs whose parents were checked for myostatin mutation were used for comparisons. All lambs were weighed at birth with a 10 g precision weighbridge in the first 24 h. The lambs were approximately weaned at 120 d of age and weighed with a 50 g precision on a platform scale. The wither height, body length, chest circumference, and rump width were taken with a sheep-type measurement stick, calliper, and tape (Hauptner and Herberholz, Germany) at the same time. The depth and area of musculus longissimus dorsi (MLD) and backfat thickness were measured by real-time ultrasound. The processes of introgression of myostatin mutation from Texel to Ramlıç sheep and genotyping methods of second backcross lambs (53 female and 61 male) were as described in the first paper of the project (Çelikeloğlu et al., 2022).

Eighteen ram lambs with the Ramlıç, BC ${}_{2}$ ( +/- ) and Texel genotypes (six lambs each) were slaughtered in a commercial slaughterhouse at the age of about 6 months and with no statistical difference between pre-slaughter weights. Hot carcass, head, foot, skin, heart and lung, full and empty gastrointestinal tract, liver, spleen, kidney, and kidney fat were weighed, and then carcasses were chilled at + 4 ${}^{\circ }$ C for 24 h.

The pH and color parameters ( $L^{*}$ , $a^{*}$ , $b^{*}$ ) of longissimus muscle were measured at the 1st and 24th hours after slaughter using a pH meter (WTW Multi 3410) and Chroma Meter (Konica Minolta CR-400).

**Figure 1:**
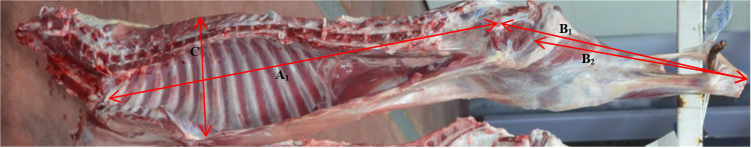
Carcass measurements ( $A_{1}$ : internal carcass length, $B_{1-2}$ : leg lengths, C : chest depth).

**Figure 2:**
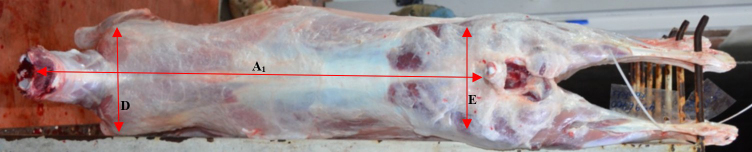
Carcass measurements ( $A_{2}$ : external carcass length, D : shoulder width, E : rump width).

Empty body weights (EBWs) of lambs were calculated after subtracting gastrointestinal content from pre-slaughter weight (PSW). Commercial dressing percentage (based on pre-slaughter live weight) and real dressing percentage (based on empty body weight) were calculated. After carcass splitting in two symmetric parts along the vertebra, cold half carcass weights (CHCWs) were recorded, and the left-half side was jointed into seven anatomical parts as described by Colomer Rocher et al. (1988): shoulder, flank, leg, neck, anterior rib, loin ribs, and tail. Each joint was weighed. The legs of lambs were dissected to divide muscle, fat, and bone and then weighed. Internal carcass length (first rib to anterior symphysis pubis), external carcass length (atlas to tail), chest depth (sternum to sixth thoracic vertebrae), shoulder width (distance between tubercle of the humerus), rump width (distance between tubercle of coxae), and leg lengths (1, anterior symphysis pubis to art. tarsometatarsal; 2, perineum to art. tarsometatarsal) were determined on the right-half carcass by using a tape measure (Figs. 1, 2).

The linear models including fixed effects of genotype, sex, birth type, month of birth, age of dam, weaning age, and weight were used to describe the collected data for growth traits and carcass characteristics by general linear model (GLM) procedure of MINITAB 18 software. All two- and three-way interactions were excluded from the models due to insufficient data in subgroups. The Tukey procedure was used for multiple comparisons.

## 3 Results

Table 1 shows the least-squares means and standard errors of growth and pre-slaughter carcass traits. The effect of genotype was statistically significant ( P<0.001 ) for all the traits except the MLD area and backfat thickness. The least-squares means and standard errors for post-slaughter carcass traits, pH and color parameters, and carcass measurements are presented in Tables 2 and 3. The commercial and real dressing percentages, pH and $a^{*}$ in first hour, external and internal carcass length, and leg lengths (1 and 2) were significantly ( P<0.05 ) affected by genotype. In addition, the influences of genotype were also significant ( P<0.05 ) on weights of half carcass and shoulder, bone and muscle weight in leg, bone and muscle ratio in the leg, weights of foot, tail, and liver.

**Table 1:** Least-squares means and standard errors for growth traits and pre-slaughter carcass traits at weaning in contemporary Ramlıç, BC ${}_{2}$ ( -/- ), BC ${}_{2}$ ( +/- ) and Texel lambs.

Trait	Genotype								P
	n	Ramlıç ( -/- )	n	Ramlıç BC ${}_{2}$ ( -/- )	n	Ramlıç BC ${}_{2}$ ( +/- )	n	Texel ( +/+ )	
Birth weight, kg	139	4.691 ± 0.114 ${}^{a}$	74	4.596 ± 0.130 ${}^{a}$	40	4.861 ± 0.151 ${}^{a}$	112	4.085 ± 0.110 ${}^{b}$	${}^{***}$
Average daily gain, kg		0.212 ± 0.005 ${}^{a}$		0.195 ± 0.006 ${}^{b}$		0.194 ± 0.006 ${}^{b}$		0.160 ± 0.005 ${}^{c}$	${}^{***}$
Weaning weight, kg		33.373 ± 0.717 ${}^{a}$		31.260 ± 0.822 ${}^{a}$		31.127 ± 0.963 ${}^{a}$		26.090 ± 0.694 ${}^{b}$	${}^{***}$
Wither height, cm	57	60.524 ± 0.788 ${}^{a}$	74	58.968 ± 0.676 ${}^{a}$	40	59.170 ± 0.706 ${}^{a}$	50	53.902 ± 0.794 ${}^{b}$	${}^{***}$
Body length, cm		58.711 ± 0.733 ${}^{a}$		57.407 ± 0.629 ${}^{a}$		57.814 ± 0.657 ${}^{a}$		52.130 ± 0.738 ${}^{b}$	${}^{***}$
Rump width, cm		14.918 ± 0.248 ${}^{a}$		14.597 ± 0.213 ${}^{a}$		14.497 ± 0.222 ${}^{a}$		13.661 ± 0.250 ${}^{b}$	${}^{***}$
Chest circumference, cm		85.14 ± 1.340 ${}^{a}$		82.53 ± 1.150 ${}^{\mathrm{ab}}$		80.87 ± 1.200 ${}^{b}$		77.05 ± 1.350 ${}^{c}$	${}^{***}$
MLD depth, cm	57	2.701 ± 0.120 ${}^{\mathrm{ab}}$	74	2.818 ± 0.103 ${}^{a}$	40	2.981 ± 0.107 ${}^{a}$	50	2.505 ± 0.120 ${}^{b}$	${}^{***}$
MLD area, cm ${}^{2}$		10.576 ± 0.581		10.195 ± 0.499		10.672 ± 0.521		9.208 ± 0.586	${}^{‡}$
Backfat thickness, cm		0.673 ± 0.025		0.630 ± 0.022		0.647 ± 0.023		0.666 ± 0.025	${}^{‡}$

${}^{‡}$ : P<0.10 ; ${}^{*}$ : P<0.05 ; ${}^{**}$ : P<0.01 ; ${}^{***}$ : P<0.001 . ${}^{\mathrm{abc}}$ : means with different superscripts in each line are significantly different ( P<0.05 ).

## 4 Discussion

In this study, the effect of genotypes on birth weight was found to be significant ( P<0.001 ), and the birth weights of Ramlıç lambs were in the range of 4.0 and 4.95 kg reported by different researchers (Bromley et al., 2000; Ceyhan et al., 2010; Demir, 1995; Fıçıcı, 2015; Hultz et al., 1935; Kaymakçı et al., 1999; Omar et al., 1992; Yalçın, 1982; Yalçın et al., 1978) in Ramlıç, Rambouillet and crosses. BC ${}_{2}$ ( +/- ) lambs tended to be heavier than the other genotypes at birth in this study. The mean birth weight of this genotype was higher than in the literature as well (Ceyhan et al., 2010; Demir, 1995; Fıçıcı, 2015; Yalçın, 1982; Yalçın et al., 1978). The introgression of the new myostatin allele into the gene pool of the Ramlıç breed may have resulted in higher weights at birth. A similar trend supporting our findings was observed by Han et al. (2010) and Farhadian et al. (2012). The birth weight (4.085 kg) of Texel lambs in the study was in the range of 3.70–5.00 kg reported for pure Texel and crosses (Estevá and Picard, 1989; Khusro et al., 2005; Koritiaki et al., 2013; Maxa et al., 2005; Wuliji et al., 1995). The birth weight of Texel lambs was significantly ( P<0.05 ) lower than the other genotypes in the study.

The average daily gain (ADG) of BC ${}_{2}$ lambs was in the middle of Ramlıç and Texel ( P<0.05 ). ADG in Ramlıç and crosses were found within the limits of 0.127–0.360 kg determined by different researchers (TAGEM, 2009; Bromley et al., 2000; Hultz et al., 1935; Jackson et al., 1997; Omar and Glafiro, 1995). The finding of the current study for Texel was just behind the 0.190–0.318 kg reported by different researchers (Ali, 1999; de Vargas Junior et al., 2014; Maxa et al., 2005). The differences may have resulted from the conditions of the farm operation, weaning ages, and statistical model.

**Table 2:** Least-squares means and standard errors for post-slaughter carcass characteristics, pH and color parameters and carcass measurements of Ramlıç, BC ${}_{2}$ ( +/- ) and Texel lambs.

Traits	Genotype			P
	Ramlıç	Ramlıç BC ${}_{2}$	Texel	
Live weight at slaughter, kg	31.801 ± 0.931	33.076 ± 0.944	32.418 ± 0.743	
Empty live weight, kg	26.502 ± 0.816	27.211 ± 0.827	27.175 ± 0.650	
Hot carcass weight, kg	14.011 ± 0.594	14.525 ± 0.602	15.881 ± 0.473	${}^{‡}$
Commercial hot dressing percentage, %	44.380 ± 1.090 ${}^{b}$	44.000 ± 1.110 ${}^{b}$	48.967 ± 0.872 ${}^{a}$	${}^{**}$
Real hot dressing percentage, %	53.194 ± 0.963 ${}^{b}$	53.508 ± 0.976 ${}^{b}$	58.336 ± 0.768 ${}^{a}$	${}^{**}$
Cold carcass weight, kg	13.549 ± 0.585	14.048 ± 0.593	15.399 ± 0.467	${}^{‡}$
Commercial cold dressing percentage, %	42.930 ± 1.080 ${}^{b}$	42.560 ± 1.090 ${}^{b}$	47.482 ± 0.860 ${}^{a}$	${}^{**}$
Real cold dressing percentage, %	51.453 ± 0.982 ${}^{b}$	51.757 ± 0.996 ${}^{b}$	56.568 ± 0.783 ${}^{a}$	${}^{**}$
Head weight, kg	1.9298 ± 0.0962	1.9575 ± 0.0975	1.7551 ± 0.0767	
Skin weight, kg	2.862 ± 0.146	2.745 ± 0.148	2.438 ± 0.116	${}^{‡}$
Foot weight, kg	0.9317 ± 0.0285 ${}^{a}$	0.9025 ± 0.0289 ${}^{a}$	0.7388 ± 0.0227 ${}^{b}$	${}^{***}$
pH (1 h)	6.404 ± 0.165 ${}^{b}$	6.392 ± 0.167 ${}^{b}$	6.880 ± 0.131 ${}^{a}$	${}^{*}$
L (1 h)	29.430 ± 1.490	31.090 ± 1.510	32.220 ± 1.190	
a (1 h)	10.846 ± 0.695 ${}^{\mathrm{ab}}$	11.248 ± 0.705 ${}^{a}$	9.095 ± 0.554 ${}^{b}$	${}^{*}$
b (1 h)	3.157 ± 0.601	3.692 ± 0.610	3.050 ± 0.479	
pH (24 h)	5.670 ± 0.192	5.482 ± 0.195	5.725 ± 0.153	
L (24 h)	34.000 ± 1.860	34.000 ± 1.890	36.820 ± 1.480	
a (24 h)	12.540 ± 1.060	13.660 ± 1.070	12.414 ± 0.844	
b (24 h)	6.567 ± 0.632	7.809 ± 0.640	7.861 ± 0.504	
Rump width, cm	18.501 ± 0.637	18.098 ± 0.646	18.769 ± 0.508	
Shoulder width, cm	17.549 ± 0.909	18.829 ± 0.922	17.153 ± 0.725	
External carcass length, cm	71.300 ± 1.570 ${}^{a}$	69.890 ± 1.590 ${}^{a}$	64.500 ± 1.250 ${}^{b}$	${}^{**}$
Internal carcass length, cm	63.405 ± 0.868 ${}^{a}$	63.382 ± 0.880 ${}^{a}$	60.519 ± 0.692 ${}^{b}$	${}^{*}$
Leg length (1), cm	38.631 ± 0.601 ${}^{a}$	38.566 ± 0.609 ${}^{a}$	35.961 ± 0.479 ${}^{b}$	${}^{*}$
Leg length (2), cm	31.519 ± 0.753 ${}^{a}$	30.422 ± 0.763 ${}^{\mathrm{ab}}$	28.692 ± 0.600 ${}^{b}$	${}^{*}$
Chest depth, cm	24.424 ± 0.722	25.110 ± 0.732	24.867 ± 0.575	

${}^{‡}$ : P<0.10 ; ${}^{*}$ : P<0.05 ; ${}^{**}$ : P<0.01 ; ${}^{***}$ : P<0.001 . ${}^{\mathrm{abc}}$ : means with different superscripts in each line are significantly different ( P<0.05 ).

**Table 3:** Least-squares means for organ weights and carcass joints (weight and ratio) of Ramlıç, BC ${}_{2}$ ( +/- ) and Texel lambs.

Trait	Genotype			P
	Ramlıç	Ramlıç BC ${}_{2}$	Texel	
Heart–lung weight, kg	1.017 ± 0.111	0.909 ± 0.113	0.8072 ± 0.0885	
Gastrointestinal tract (with content), kg	8.516 ± 0.522	9.189 ± 0.530	8.051 ± 0.417	
Gastrointestinal tract (no content), kg	3.217 ± 0.181	3.324 ± 0.184	2.808 ± 0.145	
Liver weight, kg	0.6801 ± 0.0332 ${}^{a}$	0.6683 ± 0.0336 ${}^{a}$	0.5533 ± 0.0264 ${}^{b}$	${}^{*}$
Spleen weight, kg	0.06040 ± 0.0118	0.06950 ± 0.0120	0.04230 ± 0.0094	
Testicles weight, kg	0.2392 ± 0.0539	0.2462 ± 0.0547	0.2827 ± 0.0430	
Kidney weight, kg	0.0962 ± 0.0130	0.0966 ± 0.0131	0.0951 ± 0.0103	
Kidney fat weight, kg	0.0912 ± 0.0232	0.0993 ± 0.0236	0.0473 ± 0.0185	
Right-half carcass weight, kg	6.555 ± 0.336 ${}^{b}$	6.988 ± 0.341 ${}^{\mathrm{ab}}$	7.679 ± 0.268 ${}^{a}$	${}^{*}$
Left-half carcass weight, kg	6.991 ± 0.342	6.988 ± 0.347	7.468 ± 0.273	
Foreleg weight, kg	1.3325 ± 0.0718 ${}^{b}$	1.3862 ± 0.0728 ${}^{\mathrm{ab}}$	1.5668 ± 0.0573 ${}^{a}$	*
Neck weight, kg	0.4765 ± 0.0535	0.4993 ± 0.0542	0.4071 ± 0.0427	
Flank weight, kg	0.7989 ± 0.0713	0.7111 ± 0.0723	0.8214 ± 0.0568	
Shoulder weight, kg	0.6361 ± 0.0920	0.5921 ± 0.0933	0.6526 ± 0.0734	
Back-loin, kg	1.1369 ± 0.0686	1.1490 ± 0.0695	1.2403 ± 0.0547	
Leg weight, kg	2.463 ± 0.134	2.512 ± 0.136	2.621 ± 0.107	
Tail weight, kg	0.08688 ± 0.009 ${}^{a}$	0.06890 ± 0.009 ${}^{\mathrm{ab}}$	0.05482 ± 0.007 ${}^{b}$	${}^{*}$
Leg muscle weight, kg	1.7252 ± 0.0965 ${}^{b}$	1.8288 ± 0.0978 ${}^{\mathrm{ab}}$	2.1151 ± 0.0769 ${}^{a}$	${}^{*}$
Leg bone weight, kg	0.6226 ± 0.0302 ${}^{a}$	0.5927 ± 0.0306 ${}^{a}$	0.4945 ± 0.0240 ${}^{b}$	${}^{*}$
Leg fat weight, kg	0.0933 ± 0.0192	0.0926 ± 0.0194	0.0522 ± 0.0153	
Leg muscle ratio, %	70.500 ± 2.110 ${}^{b}$	72.910 ± 2.140 ${}^{b}$	80.880 ± 1.680 ${}^{a}$	${}^{**}$
Leg bone ratio, %	24.778 ± 0.717 ${}^{a}$	23.489 ± 0.727 ${}^{a}$	18.979 ± 0.572 ${}^{b}$	${}^{***}$
Leg fat ratio, %	3.735 ± 0.867	3.623 ± 0.879	1.935 ± 0.691	
Foreleg ratio, %	19.118 ± 0.830	19.772 ± 0.841	21.005 ± 0.662	
Neck ratio, %	6.706 ± 0.688	7.096 ± 0.697	5.439 ± 0.548	
Flank ratio, %	11.308 ± 0.662	10.122 ± 0.671	11.021 ± 0.527	
Shoulder ratio, %	9.130 ± 1.03	8.520 ± 1.05	8.620 ± 0.823	
Back-loin ratio, %	16.392 ± 0.760	16.572 ± 0.771	16.680 ± 0.606	
Leg ratio, %	35.307 ± 0.976	35.954 ± 0.990	35.099 ± 0.778	
Tail ratio, %	1.217 ± 0.108 ${}^{a}$	0.984 ± 0.110 ${}^{\mathrm{ab}}$	0.7336 ± 0.0863 ${}^{b}$	${}^{*}$

${}^{‡}$ : P<0.10 ; ${}^{*}$ : P<0.05 ; ${}^{**}$ : P<0.01 ; ${}^{***}$ : P<0.001 . ${}^{\mathrm{abc}}$ : Means with different superscripts in each line are significantly different ( P<0.05 ).

The mean weaning weights of Ramlıç and BC ${}_{2}$ lambs were significantly ( P<0.05 ) higher than that of Texel lambs and not significant from each other. The findings for Ramlıç and BC ${}_{2}$ lambs were also higher than the results of the literature (Demir, 1995, 1989a; Fıçıcı, 2015; Yalçın, 1982; Yalçın et al., 1978). It is also closely similar with the range of 32.7–36.4 kg reported by Bromley et al. (2000) in Columbia, Polypay, Rambouillet, and Targhee breeds. Some researchers (Khusro et al., 2005; McEwan et al., 1988; McMillan et al., 1988; Wuliji et al., 1995) reported that the weaning weights for pure Texel and its crosses were between 20.9 and 34.42 kg. The mean weaning weight of 26.09 kg for Texel in the current study was in the range of the above literature.

No scientific reports were published for body and ultrasound MLD measurements of Ramlıç lambs at weaning. These are the first findings in the breed so far. BC ${}_{2}$ lambs attained the level of pure Ramlıç lambs in all body measurements. Wolf and Jones (2007) have reported that the body length, wither height, chest circumference, and rump width of British Texel lambs aged 140 d were 58.0, 55.8, 80.9, and 24.6 cm, respectively. These findings are closely similar to our results except for rump width. The chest circumference of Texel crosses was found to be 61.6 cm by Koritiaki et al. (2013), and this value was lower than that of our study. The results of the current study showed that the longissimus muscle depth of BC ${}_{2}$ ( +/- ) lambs was higher than the Ramlıç and Texel lambs. Likewise, Kijas et al. (2007) and Masri et al. (2011) were found that myostatin carrier lambs had deeper and larger longissimus than non-carriers. On the other hand, the result of Johnson et al. (2009) for longissimus depth was not in agreement with us and the above literature. Milerski et al. (2006) and Štolc et al. (2014) reported that mean muscle depth was between 20.71 and 26.18 mm in Texel lambs at approximately 100 d of age. Wolf and Jones (2007) found ultrasonic MLD depth of 27.5 mm at the age of 140 d in British Texels. These results were consonant with the findings of the current research.

The slaughter was carried out at the end of the research. The highest commercial and real dressing percentages were observed in Texel lambs. The differences between Ramlıç and BC ${}_{2}$ ( +/- ) genotypes were not significant in these traits. Johnson et al. (2009) and Masri et al. (2011) reported similar nonsignificant differences in a single-copy myostatin carrier and noncarrier lambs. Some non-carcass traits such as head, skin, foot, and visceral organ weights remained higher in BC ${}_{2}$ lambs. This can be the reason for higher dressing percentages in Texel lambs. When the ultimate (24 h) pH and meat color ( $L^{*}$ , $a^{*}$ , and $b^{*}$ ) parameters were examined, there were no significant differences among the genotypes. Blasco et al. (2019) deduced that the same production and slaughterhouse conditions and similar pH values might partially explain the lack of differences in the color of meat. This is consistent with the findings of the current study.

The differences in external and internal carcass lengths for genotypes were found to be significant ( P<0.05 ). External carcass length in pure Ramlıç lambs was 1.410 and 6.800 cm longer than BC ${}_{2}$ ( +/- ) and Texel lambs. The same values were 0.023 and 2.886 cm for internal carcass length. No significant differences were found between Ramlıç and BC ${}_{2}$ lambs, but there was a shortening tendency in carcasses. Johnson et al. (2009) reported that this can be due to the partially recessive effect of myostatin. The legs in the carcass of Ramlıç and BC ${}_{2}$ lambs were longer and lighter than that reported by Demir (1989b). The differences may have arisen from selection throughout the years and the absence of the fattening phase in our study. Ramlıç and BC ${}_{2}$ lambs were also leggier than Texel lambs ( P<0.05 ). It was observed that the left-leg weight of BC ${}_{2}$ ( +/- ) lambs tended to be higher than Ramlıç lambs. The differences between genotypes in muscle weight and ratio in the leg were found to be significant ( P<0.05 ). Texel lambs were higher than the other two genotypes in these traits, whereas bone weight and ratio in Texel lambs were significantly ( P<0.05 ) lower than the others. Some desirable improvements were observed in BC ${}_{2}$ lambs in carcass characteristics (Tables 2, 3). Myostatin mutation may be one of the reasons for this fact. Consequently, different researchers (Clop et al., 2006; Hadjipavlou et al., 2008; Han et al., 2010; Haynes et al., 2013; Hickford et al., 2010; Hope et al., 2013; Johnson et al., 2009; Kijas et al., 2007; Masri et al., 2011) reported that having a single or double copy of myostatin mutation results in leaner or muscled carcasses.

## 5 Conclusions

Based on the results of the present study the effects of factors such as genotype, sex, birth type, age of dam, weaning age, and birth weight on pre- and post-weaning growth characteristics, body measurements, and ultrasonic carcass traits are important. These factors should be taken into consideration in selection programs. BC ${}_{2}$ phase has been attained by marker-assisted introgression. BC ${}_{2}$ lambs were superior to Texel lambs for growth traits, while no significant differences with the maternal line (Ramlıç) were detected. So, myostatin mutation has been introgressed into the Ramlıç gene pool without harmful effects on the breed performance. The BC ${}_{2}$ lambs with deep longissimus muscle were promising. The carcass characteristics of these lambs tended to be superior to Ramlıç lambs. However, the mutation should be homozygous for commercial breeders to make maximum profit and produce leaner lambs. Therefore, selection for double-copy myostatin mutation must be carried out by a special breeding program including molecular technics. It would be useful to take into account the myostatin mutation (g + 6723G > A) as a marker for genetic progress in meat quality and quantity.

## Data Availability

Data will be made available upon reasonable request.
